# Characteristics of urban park recreation and health during early COVID-19 by on-site survey in Beijing

**DOI:** 10.1038/s42949-023-00110-3

**Published:** 2023-06-06

**Authors:** Lei Cao, Yan Sun, Angela Beckmann-Wübbelt, Somidh Saha

**Affiliations:** 1 The Key Laboratory for Silviculture and Conservation of Ministry of Education, Key Laboratory for Silviculture and Forest Ecosystem of State Forestry and Grassland Administration, Research Center for Urban Forestry, Beijing Forestry University, Beijing, China; 2 Institute for Technology Assessment and Systems Analysis (ITAS), Karlsruhe Institute of Technology (KIT), Karlstr. 11, 76133 Karlsruhe, Germany; 3 Institute for Geography and Geoecology (IfGG), Karlsruhe Institute of Technology (KIT), Karlstr. 12, 76131 Karlsruhe, Germany

**Keywords:** Urban ecology, Ecosystem services, Sustainability

## Abstract

The positive health effects of green space have received increasing attention, however, on-site surveys and city-level research to reveal the relationship between urban park recreation and urbanite health in metropolitan areas during a post-pandemic period are lacking. We conducted an on-site survey using a questionnaire with 225 respondents from 22 urban parks distributed across the metropolitan area of Beijing during the early COVID-19 eased period with another 1346 respondents in 2021 to make verification. We identified factors that could influence public perceptions of park quality and human health (i.e., physical, mental, and social health) and revealed gender differences in perceptions of park characteristics. The correspondence pattern of perceived urban park quality with social health is distinct from that of physical and mental health. Due to the strict social distancing policy in early COVID-19 period, urban parks in different levels of urbanization environment could exert varied health effects.

## Introduction

Recreation in urban parks is associated with increasing physical activity, alleviating anxiety, and promoting community identity, thereby contributing to park visitors’ physical, mental, and social health^1–3^. The beneficial relationship between green space exposure and urbanite health has received increasing attention in academic and government sectors.

World Health Organization’s Health 2020 policy framework has set green space as the prior area of creating ‘supportive environments and resilient communities^4^. A bibliometric review of 18,961 research publications on links between green spaces and public health from 1901 to 2019 found that since the 1990s there have been a steady quantitative increase in active journals, publications as well as involved institutions and countries^5^. At the beginning of 2020, coronavirus disease 2019 (COVID-19) spread globally, and the outbreak of this public health emergency changed peoples’ daily lives, particularly outdoor activities, due to social distancing policies^6,7^. Confronted by the global pandemic of COVID-19, China implemented strict containment measures^8^. An extensive concern for personal health conditions accompanied the sudden changes in daily life, and a substantial number of people suffered from severe mental health problems, such as anxiety, negative attitude toward work, and fear of loneliness^9^. Under such circumstances, access to high-quality public open spaces, such as elaborately designed urban parks, could play a primary role in improving urbanites’ health^10^.

Previous studies revealed a strong demand for external green space despite social restrictions during the COVID-19 pandemic^11^. The criteria and variables typically comprise general physical and mental health and the social aspects of health, and some research inquiries about exercise levels in the past month^12,13^. Recent research using mobile tracking data indicated that pedestrian activity increased in peri-urban forests and city parks of Norway during the lockdown period and underlined the value of urban nature as resilient infrastructure during times of crisis^14^. However, research on health-based urban park quality planning and sustainable urban development, especially for public health crisis mitigation is scarce^15^. Therefore, further understanding of the characteristics of urban park recreation and their health effects during the COVID-19 pandemic is critical for sustainable urban development.

Previous studies have often used self-reported data to evaluate individuals’ health status^16–20^. On-site interviews and paper-based questionnaires administered with urban park visitors for perceived urban park quality and health report during the COVID-19 pandemic are difficult and deficient^21^. However, on-site paper-based surveys can sample hard-to-reach respondents with low internet penetration such as senior citizens^22^. Moreover, in-person paper questionnaire survey can reach low-income respondents who might be less likely to join online research panels, which is essential to examine sustainable development issues in cities of developing countries. In addition, face-to-face surveys conducted in a park avoid respondents searching for ideas online rather than reporting on their own perception. Trained interviewers can assist respondents during on-site surveys in understanding hard questions and check each completed questionnaire in situ^23,24^. Furthermore, during on-site survey, interviewers might get direct quality comments and improvement suggestions from urban park visitors, which might help combining scientific mechanism research and park planning policy improve urban park quality^25,26^.

The megacities in urban agglomerations are considered to be the engines of future economic development in the new urbanization era of China; however, such cities have a fast-paced working mode and demanding life pressures^27^. China’s capital city, Beijing, experienced rapid urbanization^28^ and faces the challenges of “Big City Disease” such as air pollution, traffic congestion, noise, and unaffordable housing^29–31^. People living in such large cities might be at high risk of adverse effects of urbanization on human health^32^. The Beijing government launched a series of initiative to increase the provision of urban parks, and park area reached 357.2 km^2^ with 16.6 per capita at the end of 2020 (Beijing Bureau of Statistics, 2021). Scientific research efforts are required to strengthen our understanding of the physical, mental, and social health effects of urban park recreation in metropolitan areas of Beijing^33^. The establishment of guidelines for the sustainable development of Beijing, especially urban park construction, is a priority in the new development era^34^.

Although much is known about therapeutic effects of urban green spaces, there is insufficient understanding of the relationship between park characteristics and visitors’ physical, mental and social health with different recreation pattern during COVID-19 period. Therefore, this study aimed to provide insights for health-based urban park quality improvement and practical planning suggestions for sustainable urban development. We selected 22 urban parks in the most heavily urbanized region of Beijing for an on-site questionnaire survey. The remainder of this paper is structured as follows. Section 2 introduces the study area, questionnaire design, survey procedures and data analysis methods. Section 3 describes the respondents’ traits, correspondence patterns of park quality and health effects, and ordinal logistic regression results. Section 4 discusses our findings, compares with previous studies, points out planning implications for future heath crises mitigation and summarizes the limitations of the study. Section 5 concludes the study.

## Results

### Respondents’ profile and cross-district recreation pattern

The study population was concentrated in the five districts of Dongcheng, Xicheng, Chaoyang, Haidian, and Fengtai (sorted according to the proportion of the number of respondents from high to low). The proportion of males and females in the study sample was comparatively balanced, with slightly more women present (55.32%). The sample is divided into two groups: young people (15–50 years old), accounting for 56.17%, and older people (over 50 years old), accounting for 39.57%.

**Fig. 1:**
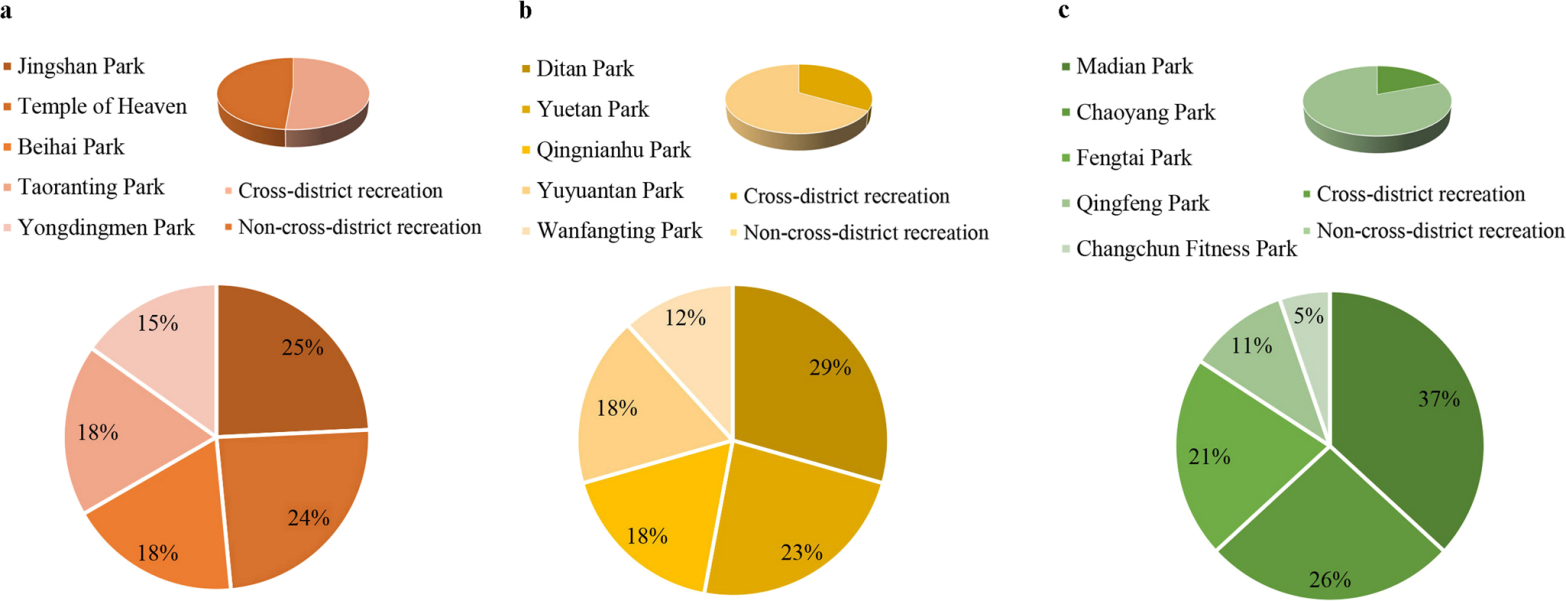
Cross-district recreation patterns in study area. **a** The proportions of top five parks in the areas within the 2nd Ring Road. **b** The proportions of top five parks in the areas between the 2nd and 3rd Ring Roads. **c** The proportions of top five parks in the areas between the 3rd and 4th Ring Roads.

The results demonstrated that during the early eased pandemic period, cross-district recreation increased with the increment in the urbanization level (Fig. 1). The cross-district recreation rate in parks of R34 was the lowest (19.1%). However, the cross-district percentage grew to approximately 51.4% in heavily urbanized areas (R2). More than half of the respondents were from other districts in Beijing. The ratio in the areas of R23 was approximately 33.3%. The top three parks with the highest cross-district proportion in those three areas were the Jingshan, Ditan, and Madian.

### Perception difference for parks in varied urbanization level

Beijing is a large-scale city with a decreasing urbanization level from the central area to the outer region, typically expanding in a concentric circle. Therefore, we analyzed the parks in regions R2, R23, and R34 to examine the influence of urbanization on perceptions of park characteristics (Supplementary Table 2). Regarding the perceptions of spaciousness and cleanliness, visitors in R2 showed significant differences from those in R23 and R34, evaluating the parks in R2 as cleaner and more spacious. Moreover, visitors in R2 recognized the disparity between safety and park maintenance compared to R34. For those parks in the heavily urbanized area (R2), the median perception value of park recreationists was 9, which decreased to 8 in R23, and rebounded to 9 in R34. The average value for parks in R2 was the highest, with 8.91, 7.86, and 8.32 for R2, R23, and R34, respectively. However, for perceptions of the quantity and quality of fitness equipment, urban parks in R34 gained the highest evaluation. In addition, we analyzed variation in perceptions of park landscape and ecosystem services from the urban center to outer areas. The perceptions of greenery coverage and tree diversity for parks in R2 significantly differed (higher evaluations) from those in R23 and R34. The median perception of cultural value in R2 was 9, which was significantly higher than the 7 and 6 scores in R23 and R34, respectively. Although the perception of intimate nature experiences showed the same median value of 8, there was a decreasing evaluation pattern from the urban center to outer areas. The evaluation of entertainment value did not show significant differences between parks in R2 and R23; however, the parks in the urban center (R2) were perceived as more entertaining than the parks located in the outer areas (R34).

### Perception difference in respondents with varied park access

To explore the influence of accessibility on park quality perceptions and health, we classified the respondents into those who can only access one park from their home, and those who can reach at least two parks on foot (Supplementary Table 2). The comparison results revealed that respondents who could access more parks gave higher evaluations for cleanliness, overall quality, landscape uniformity, and flower and grass proportions than those who could reach only one park. Moreover, we analyzed the preferences of respondents who could go to multiple parks and found a disparity between the young to middle-aged (15–50) and older people (>50) for park choices. Community parks, such as Changchun Fitness Park and Qingfeng Park, were the most popular among young and middle-aged visitors, whereas older visitors preferred scenic parks, such as Beihai Park and Xuanwuyiyuan Park.

### Perception difference in respondents’ age and gender

Both young and older groups demonstrated significant differences in tree diversity and air purification with older people showing greater appreciation than young people (Supplementary Table 2). There were significant differences in quality characteristics between men and women, indicating the influence of gender on perceptions of park characteristics. Women evaluated overall quality, park maintenance, lawn and flower proportion, tree species richness, and intimate nature experiences in the park higher than men. Notably, there were differences based on gender but not age for park management category.

We divided respondents into four classes according to age and gender (young man, older man, young woman, and older woman) to examine the demographic differences in park quality perceptions (Supplementary Table 3). Significant differences occurred in the evaluations between young women and men in park maintenance, overall quality, lawn and flower proportion, and tree diversity. For park maintenance, the median perception value was 9 for young women and 8 for young men. For lawn and flower proportion and tree diversity, the median perception value was the same 8, and the average values of young women (7.76 and 7.72) were higher than those of young male populations (8.36 and 8.32). Both young and older females gave significantly different evaluations of amenities (e.g., facility to nurse babies and toddlers) and air purification functions. Amenities only scored a median value of 8 from young female visitors, whereas older female visitors graded 9. Regarding air purification, the median values were 9, with a higher average for older female visitors. Although young female visitors were as insensitive as young men to the air purification services of urban parks, young female visitors were dissatisfied with amenities and gave them the lowest score among the four park quality classes. The minimum number of park quality features that showed significant differences occurred among older men and women, where only one feature (i.e., entertainment services) was found.

### Correspondence pattern of perceived park quality features

**Fig. 2:**
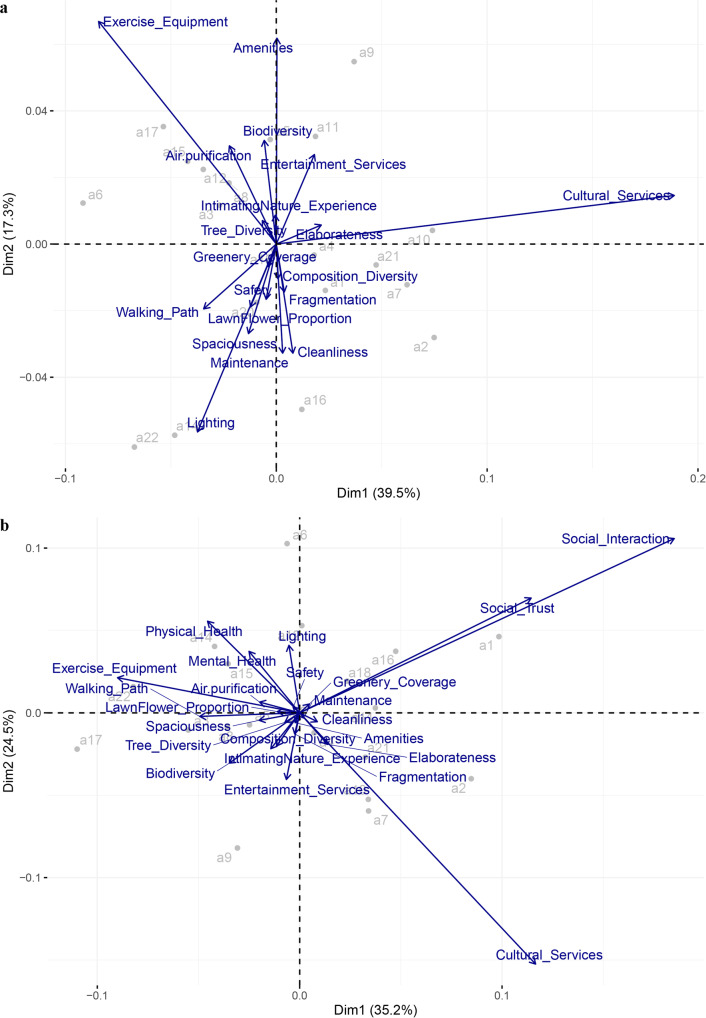
Correspondence pattern of perceived park quality features and health. **a** Biplots of the correspondence analysis of 20 urban parks in terms of park quality features only. **b** Biplots of the correspondence analysis of 20 urban parks in terms of park quality with urbanite health. Urban parks are in gray points and quality features (from Table 2) and physical, mental and social health are in blue texts. Long lines between the quality features and the origin indicate a strong association. Small angles between two lines of quality features indicate associations. Lines with angles near 180 degrees present negative associations. The distance between any urban parks shows a measure of their similarity.

The correspondence pattern of park quality features (Fig. 2a) as well as the pattern of park quality features and physical, mental and social health (Fig. 2b) are analyzed. First, for park quality features, the CA biplot illustrates the first two dimensions, which together explained 56.8% of the total variances in park quality features (Fig. 2a). There was a small angle between quality features of “Lighting” and “Walking_Path”. The variables “Maintenance” and “Cleanliness” show similar trajectories in the CA biplot. There was either a weak or no correlation between “Exercise_Equipment” and “Cultural_Services” or “Lighting”. Most quality features did not show strong association, indicated by the short lines to the origin. The quality features that belonged to the same category showed similarities, indicated by their short distance, such as “Maintenance” and “Cleanliness”. In addition, according to the evaluations of urban park quality from all respondents, the CA biplot showed the similarities of surveyed urban parks, such as the high similarity between a7, a10, and a21. Second, the self-reported social interaction frequency and trust demonstrated strong similarities, but self-reported social health aspects presented weak or association with physical or mental health (Fig. 2b). The physical and mental health showed high similarity, indicated by the small angle of lines and close distances between two texts. High “Exercise Equipment” evaluation is closed related with “Physical health” and “Mental health”. Although the lighting feature might be moderately associated with mental health, indicated by the short lines to the origin. It is noteworthy that ‘Cultural Services’ and ‘Physical Health’ presented negative associations, indicated by the near 180 degrees angles of two lines.

### Urban park clustering based on self-reported health

**Fig. 3:**
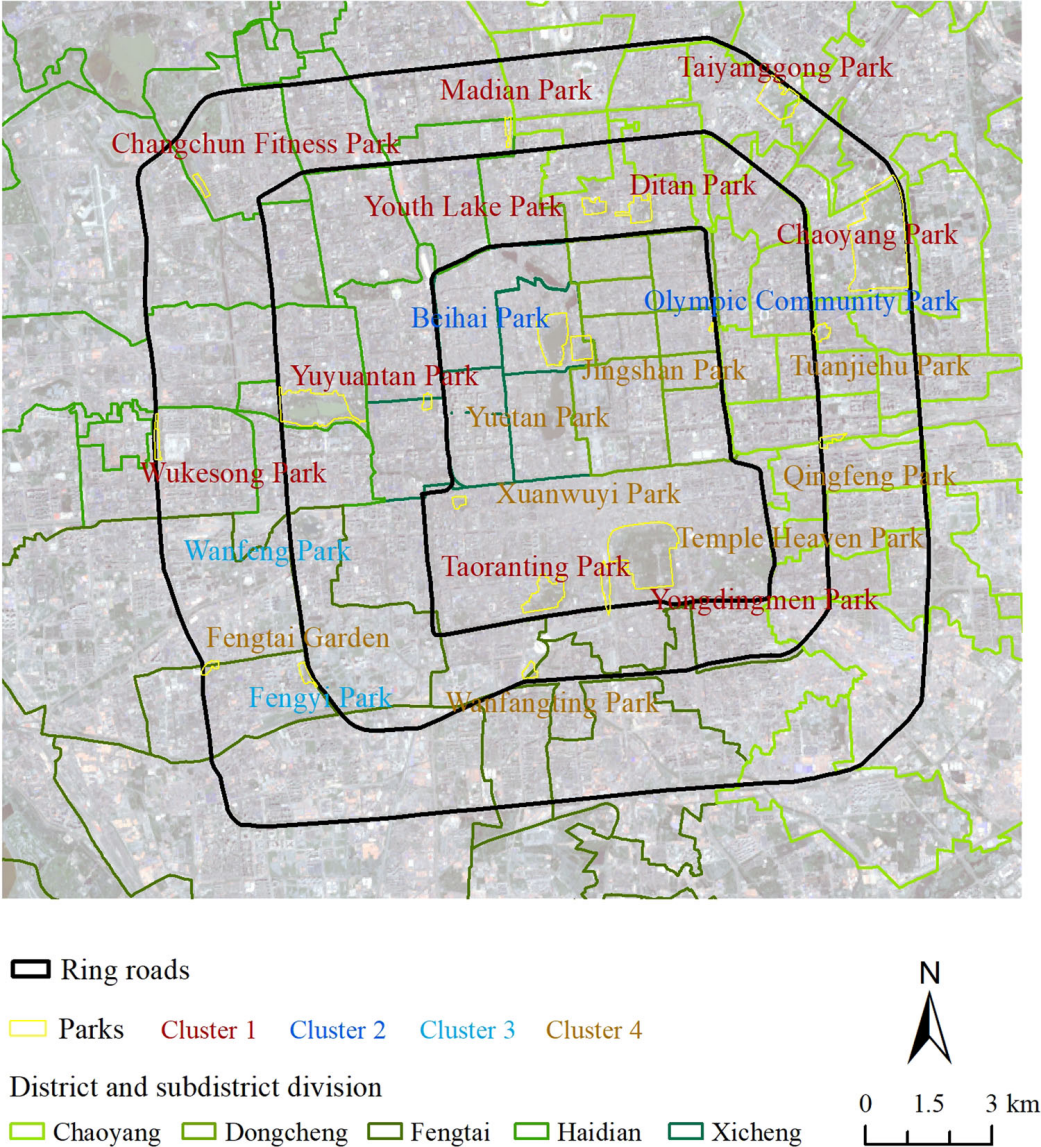
The distribution of urban park clustering by the health effect of recreation. Urban parks are classified into four clusters in different colors. Rings roads, districts and subdistrict division in Beijing metropolitan area are illustrated by lines. The boundary of urban park is in yellow color.

We classified the 22 parks into four groups using k-means clustering, and found that the group of parks based on respondent’s self-reported health scores differed from the classification based on geographical position. The first group exhibited the worst social health, the second group had the best social health values, the third group showed the highest physical and mental health scores, and the last group presented moderate social, physical, or mental health conditions (Fig. 3). It is noteworthy that only two parks (Fengyi and Wanfeng Park) are classified in the third group. Both parks are community parks built by the local government of Fengtai District. Most scenic parks, such as the Temple Heaven Park were not clustered into a high health featured group, except Beihai Park, which demonstrated a high social health score. The Olympic Community Park belonged to the same class as Beihai Park.

### Factors influencing health and park quality perceptions

We found varied influencing factors on physical, mental, and social health. First, only age of respondents was related to variations of physical health (Supplementary Table 4). Among three age groups, the OR value for young visitors with high self-reported physical health was the highest. Second, gender was significant for mental health, with male visitors reporting negative conditions. For social health, we collected data regarding social interaction and trust and found that only age factor was significant for social interaction and the negative effect indicated that elderly might present better social interaction. All significant outcomes of the ordinal logistic regression for human health are presented in Supplementary Table 4. None of the variables showed significant influence on physical activity level or social trust. For park quality perception (Supplementary Table 5), 12 characteristics were significantly affected by of urbanization level, gender, age, or access to parks. None of the variables in the category of infrastructure quality perceptions (walking path, lighting, exercise equipment and amenities) were significantly influenced by demographic and park environment factors. It is noticeable that access was significantly correlated with cleanliness, safety, overall quality, fragmentation, lawn and flower proportion, and tree diversity, and access to a single park or no accessible park exerted a negative impact on perceptions of each urban park quality feature. In addition, the urbanization level of the park locations had a positive effect on park quality evaluation, indicating that urban parks located in the area within the Second Ring Road likely earned higher perceptions. The OR value of heavy urbanization level varied from 0.651 to 2.099, which pattern was higher than other independent variables included in the logistic regression. All significant outcomes of the ordinal logistic regression for park quality perception are presented in Supplementary Table 5.

## Discussion

Among accessible park number, gender, age, and urbanization intensity level, only gender was found to be significantly related with mental health and males were less likely to show high self-report. But age was detected to be significant influencing factor for both physical and social health during the COVID-19 pandemic. While a similar study in Chengdu, a major city of China, did not detect significant differences in terms of physical health and social interaction during the pandemic^35^. The varied health effects of urban park recreation might be attributed to the demography differentiation in park quality perception of Beijing metropolitan area. Our results have revealed the sensitivity of gender on perceptions of park characteristics and females tended to give higher evaluation than males during the COVID-19 pandemic. The observed gender difference is consistent with previous findings^36,37^, but in contrast with the findings with no gender differentiation^38^. While for urban park infrastructure quality (walking path, lighting, exercise equipment and amenities) perception, we found no significant relationships across gender and age groups during the COVID-19 pandemic.

The study was conducted in October and November 2020, when the first round of the COVID-19 epidemic in China had been controlled, and short- and medium-distance urban park recreation became an increasingly popular choice of recreational weekend activities^11^. Although the overall quality of urban parks is better in areas with higher urbanization intensity, visitors in parks with lower urbanization intensity showed higher self-reported physical health. This is consistent with a previous study on the population groups aged over 60 of Shanghai that higher community urban density was related to lower self-reported life satisfaction^39^. Future research on the physical health effects of urban green space recreation should not only assess the park use pattern, but also take the parks’ social background and context (urbanization level, construction history and government planning prospect) into consideration. However, there was non-significant mental health difference in areas with varied urbanization level, indicating the complicated impact of green space on human health during the COVID-19 pandemic. A study of 577 urban parks in Nanjing, a major city of China revealed that the COVID-19 pandemic awakened Chinese residents’ desire for high-quality urban parks^40^. Our results showed that during COVID-19 pandemic eased period, the cross-district recreation proportion was highest in the regions within the 2nd Ring Road where most high evaluated urban parks are located. As an ancient capital, the central area of Beijing owns many historic scenic parks (e.g., the Jingshan Park) with high financial budget from local government and well-designed management and conserve systems. The correspondence analysis in the study demonstrated this city’s distinct cultural services during the COVID-19 pandemic^41^. While cross-district park recreation could also be driven by nearby shopping malls, museums, or restaurants^42^. Hence, we emphasize that city-level analysis on physical and mental health effects of park recreation should pay much attention to the parks’ background and context (urbanization level, construction history and maintenance priority), especially in metropolitan areas of China. A recent study in Rio de Janeiro, the second largest city of Brazil found that compared with parks and green views, home gardens were the most efficient type of urban greens infrastructure in mitigating mental distress during the current COVID-19 outbreak^17^. Therefore, the confinement policy and urbanization background could combine to influence the health effects of urban park recreation during public health crises.

Urban parks work as the pathway to a consoling effect of nature upon people struggling in rowdy and hurried urban lives in cities^43,44^. As one of the only sources of resilience amidst the coronavirus pandemic, urban parks’ refuge service should be emphasized to improve urban resilience to public health stress^14^. The present study revealed the planning potential for using urban parks to promote the city’s urban development, especially for those megacities in developing countries.

The correspondence analysis illustrated that the relationship between urban park quality and physical or mental health could be different from that of social health. Planning practice in lighting, exercise equipment, walking path, air purification function of urban park should be paid much attention in Beijing. Although many investigations have been carried out to explore the health effects of urban park use on physical or mental health during the COVID-19 pandemic, more are needed to clarify the mechanisms underlying the differentiations in perceived physical, mental and social benefits of urban park recreation in megacities^45^. A study in New York based on 138 racially diverse participants revealed the social-spatial disparities in sense of belonging during the pandemic^3^. We suggest that elaborate planning practice based on cultural differences should be integrated into urban park quality improvement to enhance the social well-being benefits of urban park use in metropolitan areas of China^46^.

In addition, our results implied that despite the importance of landscape configuration patterns in landscape ecology, landscape composition traits such as tree diversity, greenery coverage and flowers and lawns proportion might be effective factors to enhance the benefits perceived by sensitive population groups during the COVID-19 pandemic^47,48^. Along with the consistent increase of urban park acreage in Beijing, park management and infrastructure could not lag behind. Particular attention should be paid to park safety, convenient childcare facilities, walkway quality, or specialized devices for those with reduced mobility, in order to support sustainable urban park provision^10,49^. Furthermore, our findings underscore the urgency of incorporating environmental justice into urban park planning and management for mitigating COVID-19 and other future health crises^26,39,50^. Along with the rapid economic development of megacities in developing countries, it is not only the value of natural capital that should not be overlooked, but gender- and age-prioritized design should be a key element to promote environmental justice^51,52^. Our results found that visitors with more accessible parks in Beijing gave higher evaluations on park quality and landscape traits, such as park cleanliness and landscape fragmentation than those with only one or no accessible park nearby. The negative effect of green deprivation and esthetic fatigue in urban park recreation could affect visitors’ perception of park quality, since deprived groups might tend to rate parks harshly. Therefore, urban park accessibility might be one of the requisite factors to realize long-term positive feedback from the public for local greening projects^53^.

While the respondents to the on-site questionnaire covered most districts of the metropolitan area of Beijing, we lacked data from the Shijingshan District. We did not collect the education, marriage, or income status of respondents in order not to bring about visitor’s displeasure and rejections to finish all questions in such a prevalence period of anxiety and depression. While these demographic traits could be valuable for understanding the differences in self-reported satisfaction in future research^54^. The asymptote of the accumulation curve of respondents’ opinion diversity increased after ten respondents. It meant a higher number of respondents would have better captured the diversity of opinions in those parks. A larger sample size (i.e., 400 responses for each park) might better capture details and bring about more reflections, however recruitment might be challenging considering the difficulty we experienced in completing on-site questionnaires of park use and perceptions during early eased pandemic period. Under the circumstances of sparked alarm and social distancing, we call for multisectoral cooperations to facilitate green space and urbanite health research and therefore contribute to urban sustainability enhance. The on-site questionnaire was conducted on weekends, which might have caused bias since some groups of residents might prefer to visit urban parks on weekdays. The population deprived of green space in their residential region might visit urban parks on weekdays during times of stress to enjoy the benefits provided by nature. A study on weekday urban forest recreation in Bonn, Germany found that during the COVID-19 pandemic, there were novice urban park visitors (e.g., young people), and recreation culmination occurred in the late afternoon, which differed from the common peaks seen before and after office hours^55^. Longitudinal studies on the health effects of urban green space recreation are also necessary. The ecosystem services (e.g., esthetic benefits and trapping particulates) of urban green space could vary with the seasons^56^. Given these considerations, future discussions based on multi-season and temporal research will deepen our understanding of the interaction between urban green space recreation and human health. The on-site questionnaire was conducted in Beijing, the capital of China, and we did not make comparisons with another megacity or low-tier city. Multiple cities comparison or comparative studies for megacities in multiple countries could contribute to the comprehensive understanding of the interaction between urban park recreation and urbanite health. Further multi-year and longitudinal studies are required to deepen our understanding of the interaction between urban forest recreation and human health.

## Methods

### Study area

Beijing is China’s capital city with 16 municipal districts (Fig. 1a, b). We selected 22 urban parks in Beijing as the study area. These urban parks (Table 1) are all municipal parks larger than 2 ha within Beijing’s Fourth Ring Road, the central area of this megacity. The selection criteria ensured that all parks in this study had at least moderate service coverage^57^ and were included in the Statistical Yearbook of Beijing Parks and Forestry. Beijing has experienced a rapid pie-style urban space expansion since the 1978 Reform and Opening Up Policy, and the average annual growth rate of urban land areas was approximately 3.5% from 1978 to 2015^28^. To promote sustainable urbanization and enhance the wellbeing of Beijing’s citizens, the government has developed greening strategies to improve urban park quality^58^. Within the Second Ring Road, Xicheng and Dongcheng districts are the most heavily urbanized areas of Beijing, with many ancient royal gardens. The intensity of urbanization, indicated by the percentage of impervious surface area, of the regions within the Second Ring Road (R2), between the Second and Third Ring Roads (R23), and between the Third and Fourth Ring Roads decreases progressively (R34). Urban forests in these three regions with varying urbanization intensities could demonstrate distinct characteristics such as accessibility^59^.

**Table 1:** Selected urban parks (*n* = 22) in the metropolitan area of Beijing.

Administrative district	Name	Location	Area (ha)	Year of build
Xicheng				
	Xuanwuyiyuan Park	R2	7.1	1984
	Beihai Park	R2	69.1	1925
	Jingshan Park	R2	26.6	1928
	Yuetan Park	R23	7.0	1955
	Taoranting Park	R2	52.1	1952
Dongcheng				
	Youth Lake Park	R23	17.6	1960
	Ditan Park	R23	30.6	1984
	Yongdingmen Park	R2	17.3	2004
	Olympic Community Park	R2	1.36	2004
	Temple Heaven Park	R2	273.0	1420
Haidian				
	Madian Park	R34	7.3	2003
	Wukesong Olympic Park	R34	9.9	2008
	Yuyuantan Park	R23	136.7	1960
	Changchun Fitness Park	R34	10.6	2007
Chaoyang				
	Qingfeng Park	R34	9.7	2009
	Tuanjiehu Park	R34	11.7	1986
	Chaoyang Park	R34	285.1	1992
	Taiyanggong Park	R34	59.0	2002
Fengtai				
	Fengtai Park	R34	7.6	1986
	Fengyi Park	R34	9.4	2005
	Wanfeng Park	R34	78.0	2007
	Wanfangting Park	R23	9.2	1990

Due to the COVID-19 pandemic, the Chinese government implemented a policy of home isolation for approximately one month from late January 2020. The second wave of outbreak in Xinfadi food market in the summer caused more cases than the first wave. The daily activities in educational institutions, especially those in universities were hard to recover until the late September. The on-site questionnaire for this study was conducted in October and November 2020, approximately six months since the beginning of the pandemic.

### Questionnaire

The study sample was the permanent population, and we required that respondents had visited the surveyed park at least once within the previous month. The questionnaire collected demographic information (age, gender, district of residence) from respondents (Part 1), as well as information regarding their transportation mode to the park and the frequency and duration of visits per month (Part 2). The central part of the questionnaire (Part 4) inquired regarding visitors’ perceptions of park characteristics, including its maintenance condition, infrastructure provision, landscape structure, and ecosystem services^60,61^. The individual variables selected to capture the characteristics of the urban park are presented in Table 2. All respondents were requested to evaluate each of the variables on a scale from 0 to 10, where 10 is the highest possible value^54^. We present the questionnaire (Supplementary Methods) and examples of the scenes recorded by the researchers during the on-site administration of the questionnaire (Supplementary Table 1). All respondents will be informed of the nature of the research project and what their involvement will mean. The whole study process complies with national laws and the rules and regulations of Beijing Forestry University and is handled in accordance with the ethics requirement of the College of Forestry. The research procedures were reviewed and approved by the College of Forestry of Beijing Forestry University.

**Table 2:** Variables selected to capture urban park characteristics

Component	Variables	Description
Usage	Transport mode	Bicycle/e-bike/private car/public transport/walking
	Frequency	(manually fill)
	Duration	(manually fill)
Management	Cleanliness	Clean and free from dirt, trash or waste
	Safety	Acceptable level of being protected from danger
	Maintenance	Functional checks, servicing, and repairs
	Spaciousness	Visual experience and narrow feeling
Infrastructure	Walking path	Single use for walking
	Lighting	Enough light fixtures for evening visiting
	Exercise equipment	Apparatus for physical activity
	Amenities	Facilities for convenience e.g., toilet access and facility to nurse babies and toddlers, seating
Landscape structure	Elaborateness	Detailed and complicated in design and planning
	Fragmentation	Small and isolated patches
	Composition diversity	Diversity of landscape features and habitat types
	Coverage of greenery	Site coverage of green plants or branches
	Lawn and flower proportion	Provision of lawn area and flower decoration
	Tree diversity	Abundance of tree species
Ecosystem services	Air purification	Improved air quality compared to outside park
	Biodiversity	Variety of life e.g., bird, hedgehog, squirrel
	Cultural services	Historical meaning and education service
	Entertainment services	Amusement facility e.g., carousel, sliding board, swing, sightseeing boat
	Intimate nature experience	Aroused love of nature during visit

This questionnaire design asked each respondent to self-report their health conditions (Part 3). The method was based on Seligman and Csikszentmihalyi (2014)^62^, who found that subjective health might be more meaningful than objective health in living a happy life. Therefore, we used five broad questions to evaluate visitors’ health, rated on a scale from 0 to 10 (from very bad to very good), regarding overall physical and mental health^53,63^, levels of physical activity^33,64^, levels of social trust and interaction^65^, and daily variation of outdoor exercise and mood in a week and neighborhood committee involvement^66^.

### Survey procedure

In October and November 2020, a person-to-person on-site questionnaire was administered to gather data on park recreationists’ self-reported health conditions in three aspects (i.e., physical, mental and social health), perceptions of various green space qualities, and individual recreation modes during early COVID-19 period. Respondents were chosen randomly, and researchers avoided concentrated locations or distributing the questionnaires only in a large group of visitors in order to minimize bias. At least 10 questionnaires were completed in each of the 22 studied parks^60^. All data collection took place between 8 AM to 8 PM during weekends to ensure comparability and representativeness of urban park visitors. One repetitive on-site survey on the urban park recreation for verification was conducted from September to November in 2021 and we collected 1346 questionnaires from 18 urban parks. These 4 parks in Fengtai District were excluded in the survey in 2021 because of the local epidemic and controls. The questionnaire consists of the same contents as those in 2020 except Part 4, in order to obtain a larger sample size in such a time of crisis.

### Data analysis

We proposed descriptive and regression analysis with three steps. First, we examined the difference significance regarding park quality and urbanite health according to five factors (i.e., gender, age, access to park, urbanization level, and park type). The access to park refers to the number of accessible urban park from the residence by walking, obtained from question 4 in Part 2 by inquiring. Urbanization level refers to park position environment (i.e., R2, R23 and R34) in terms of urbanization intensity. The park type refers to scenic park and community park in this study and is identified according to their management agencies. Second, we analyzed the selected 22 urban parks according to health effect. Finally, we explored the effects of gender, age, and access to parks as well as park environment (urbanization level) on physical, mental, and social health. The analysis of urban park recreationists’ perceptions excluded children (0–14 years old), since most of those 10 children visited the parks based on the prerequisite of their parents accompany. We reclassified the 225 respondents (Table 3) according to the visitors’ accessible park number (i.e., only one or no accessible park and more than one park) and demographic information (gender, age, and residential district). Median and mean values were used for descriptive analyses. The differences between respondent groups were identified using the Mann–Whitney U test. We selected the nonparametric test under the condition that the distributions of the grouped data were unknown. We formed clusters for the 22 selected parks and a Hierarchical Cluster Analysis (HCA) using Ward’s minimum variance method was run on the evaluation scores of the features included in the study to calculate the distances between clusters and determine the number of clusters. Differentiated clusters extracted by *K*-means demonstrated the influence variation of urban parks in terms of physical, mental, and social health^67^. We made use of the second survey data in 2021 and conducted HCA to verify our results (Supplementary Fig. 2).

**Table 3:** Participant characteristics.

Attribute	Subgroup	Number	Percentage (%)
District of residence	Chaoyang	45	19.15
	Dongcheng	52	22.13
	Fengtai	42	17.87
	Haidian	44	18.72
	Shijingshan	0	0
	Xicheng	52	22.13
	Other	0	0
Gender	Male	105	44.68
	Female	130	55.32
Age	0–14	10	4.26
	15–25	40	17.02
	26–50	92	39.15
	51–64	44	18.72
	Over 65	49	20.85
Access to park	>1	138	58.72
	≤1	97	41.28

To analyze the influence of demographic traits, urbanization, and park recreation mode, we explored the variations in perceived physical, mental, and social health and urban park quality and their relations with certain independent variables^42^. We used ordinal logistic regression to determine whether the variables had a significant influence and extracted the odds ratio (OR). This method estimates probabilities using a logistic function^68^. Statistical analyses were performed using IBM SPSS Statistics 20.0. The ordinal logistic regression under the Generalized Linear Models in SPSS can directly give the OR values and the results will be based on the use of Likelihood ratio chi-square tests which are more powerful than the results solely based on the Wald test. The SPSS runs the ordinal logistic regression model in this way:

1 $${\rm{logit}}(P({\rm{Y}}\le j))={\beta }_{{\rm{0j}}}-({\beta }_{1}{X}_{1}+{\beta }_{2}{X}_{2}+{{\ldots }}+{\beta }_{n}{X}_{n})$$

where *Y* is an ordinal dependent variable with various categories and *j* is a specific category. The key term of logistic regression is coefficients ($\beta$) and the index OR is exp ($\beta$). Positive values of $\beta$ indicate higher odds of moving to the next higher ordered category for higher values of *X*.

The index OR reflects the multiplicative change in the odds of being in a higher category of the dependent variable for every one unit increase in the independent variable. An OR > 1 suggests an increasing probability of being in a higher category of dependent variable as independent variable values increase. An OR < 1 suggests a decreasing probability with increasing values of independent variable. An OR = 1 suggests no predicted change in the probability. Before performing ordinal logistic regression, variance inflation factor (VIF) was used to identify multicollinearities and variables that showed multicollinearities have been excluded from the model. The final models were determined mainly by three steps. First, the variables in the models passed the Test of Parallel Lines, which indicated that the proportional odds assumption was satisfied. Second, the Model Fitting Information by likelihood ratio chi-square test for each model was significant (*P* < 0.05), which revealed a significant improvement in fit of the final model over the intercept only model. Third, the corrected Akaike information criterion (AICc) was used to determine the final models with best fit. We made tables to show the regression coefficients and significance tests for each of the independent variables in the model.

**Fig. 4:**
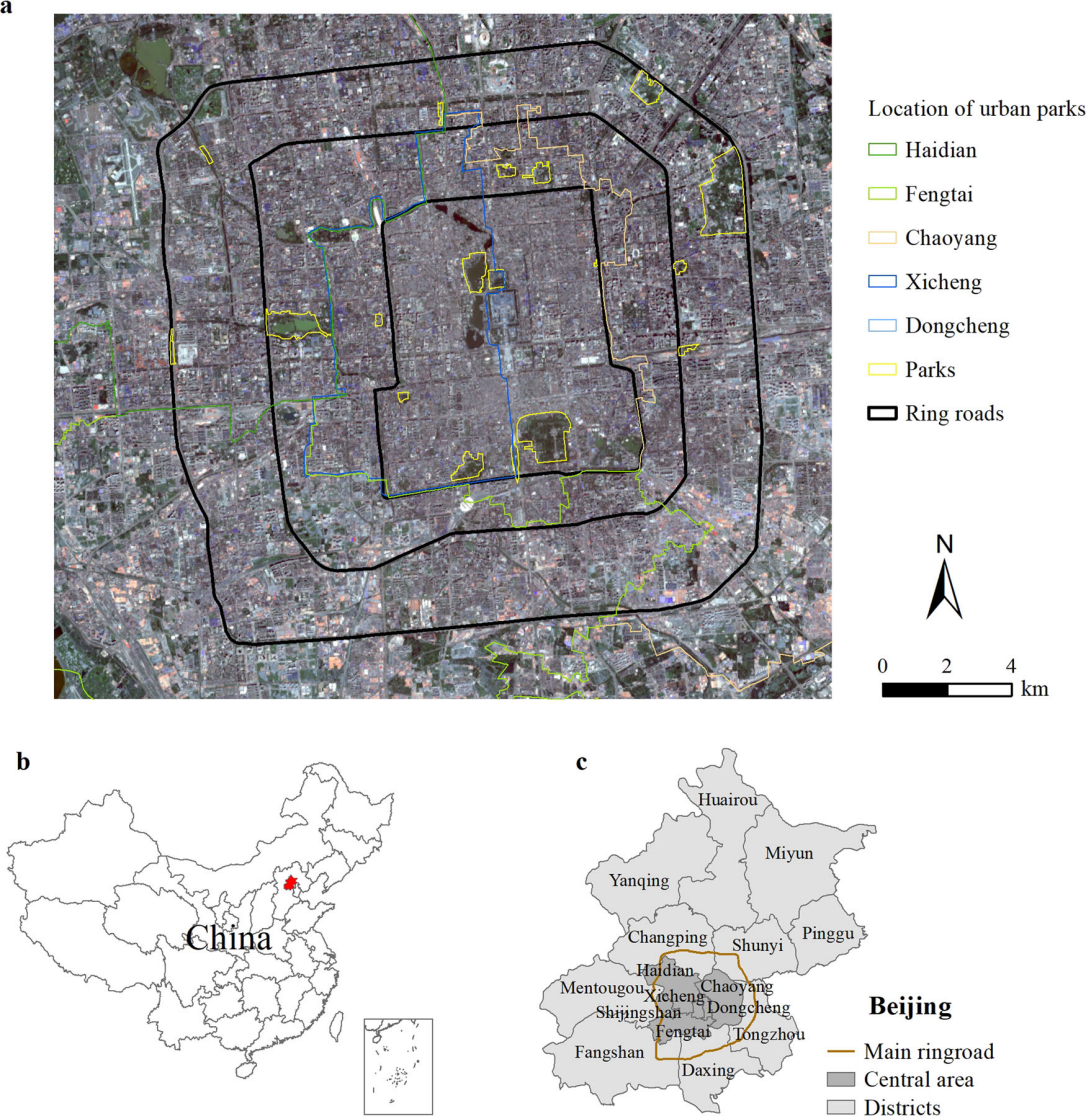
Study area. **a** Location of the 22 selected urban parks in the metropolitan area within Beijing’s Fourth Ring Road (A). **b** Beijing in China. **c** The central six districts of Beijing.

We further conducted correspondence analysis (CA) to reveal the relation between urban park quality and human health. Mathematically, CA, similar to principal component analysis, provides spatial representation based on the chi-squared distance. We used CA to demonstrate similarities between urban parks and examined whether park quality variables (Table 2) were closely related to each other. The CA biplots can illustrate the interdependence of urban park quality variables and the distribution of 22 urban parks included in this study (Supplementary Fig. 1). However, as two of the urban parks (a13 and a19) were considerably different from the other 20 parks, we mapped the other 20 urban parks again to conduct the CA. The rather poor maintenance or strip park shape of a13 or a19 might have resulted in their distinctions from other 20 urban parks. Similarly, we also mapped the correspondence pattern of urban park quality and human health using 20 parks. The distribution pattern was illustrated in Fig. 4. To avoid unnecessary disclosures, we anonymized the urban parks in the CA biplot. Specifically, the same axis domain of the park quality feature variables’ location indicated a positive clustering pattern, and a large distance between variables indicated a weak correspondence relation. Statistical analyses and plotting were performed using the R package FactoMineR^69^.

### Reporting summary

Further information on research design is available in the Nature Research Reporting Summary linked to this article.

## Supplementary information

Supplementary Materials

Reporting Summary

## Data Availability

The data that support the findings of this study are available from the corresponding author upon reasonable request.

## Code availability

No custom codes were used. The codes used to analyze the data and create the plot of this study are available from the corresponding author upon reasonable request.
