# Supplementary material for: Characteristics of urban park recreation and health during early COVID-19 by on-site survey in Beijing
Source: NPJ Urban Sustain. 2023 Jun 6;3(1):31. doi: 10.1038/s42949-023-00110-3 (PMC10243239; doi:10.1038/s42949-023-00110-3)
Supplement: Supplementary file 1 — Supplementary Materials [file 42949_2023_110_MOESM1_ESM.pdf]

Supporting Information for

Characteristics of urban park recreation and health during early COVID-19 by on-site survey in Beijing

Lei Cao, Yan Sun[*], Angela Beckmann-Wübbelt, Somidh Saha

Supplementary Methods

Questionnaire in English

Part 1 Personal information

1. Age

○ 0-14	○ 15-25	○ 26-50	○ 51-64	○ 65 以上

2. District of residence

○ Chaoyang	○ Haidian	○ Shijingshan	
○ Dongcheng	○ Xicheng	○ Fengtai	○ Other: ________

3. Gender

○ Male	○ Female

Part 2 Urban park recreation

4. How many parks are accessible from your residence by walking? ________

5. How about the perceived accessibility of parks (by walking) near your residence?

	0–10 (very unsatisfied to very satisfied)
Size of accessible parks	
Distance of accessible parks	

6. Transport mode to this park (Multiple choice)

○ Biking	○ E-biking	○ Private driving	○ Public driving	○ Walking

7. How many times per month do you visit this park? ________

8. How much time do you spend in this park? ________

Part 3 General status of self-reported health（physical, psychological, social）

9. How is your physical health?

	0–10 (very bad to very good)
How is your physical health in general?	
What is your level of physical activity?	

10. How is your mental health?

	0–10 (very bad to very good)
How is your mental health in general?	

11. How is your social cohesion and trust?

	0–10 (never to a lot)
Do you often chat with your neighbors?	
Do you trust your neighbors?	

12. How many days in a week do you …?

	1	2	3	4	5	6	7
Walk outdoors for more than 15 minutes	○	○	○	○	○	○	○
Keep a positive mood	○	○	○	○	○	○	○

13. Are you involved in a neighborhood committee?

○ Yes	○ No

Part 4 Evaluation of the park's attributes

14. How do you evaluate the general condition of this park?

	0–10 (very bad to very good)
The spaciousness (compact to spacious)	
The cleanliness	
Safety	
Maintenance	
Overall quality	

15. How do you evaluate the facilities provided by this park (quantity and quality)?

	0–10 (very unsatisfied to very satisfied)
Walking paths	
Lighting	
Exercise equipment	
Amenities (e.g., toilets, seating)	

16. How do you evaluate the green landscape of this park?

	0–10 (very plain to very elaborate)
Landscape design beauty	
	0-10 (very irregular to very unfragmented)
Landscape fragmentation	
	0–10 (very single to very diversified)
Landscape composition diversity	
	0–10 (nothing green to very green)
Coverage of greenery	
	0–10 (very few to many)
Lawn or flower proportion	

	0–10 (very low to very high)
Tree diversity	

17. How do you evaluate the ecosystem services of this park?

	0–10 (very unsatisfied to very satisfied)
Air quality compared with outside the park	
	0–10 (very low to very high)
Biodiversity (animals, plants, etc.)	
How do you appreciate the historical and cultural value of this park?	
How do you appreciate the entertainment value of this park?	
How do you appreciate the experience of being close to nature in this park?	

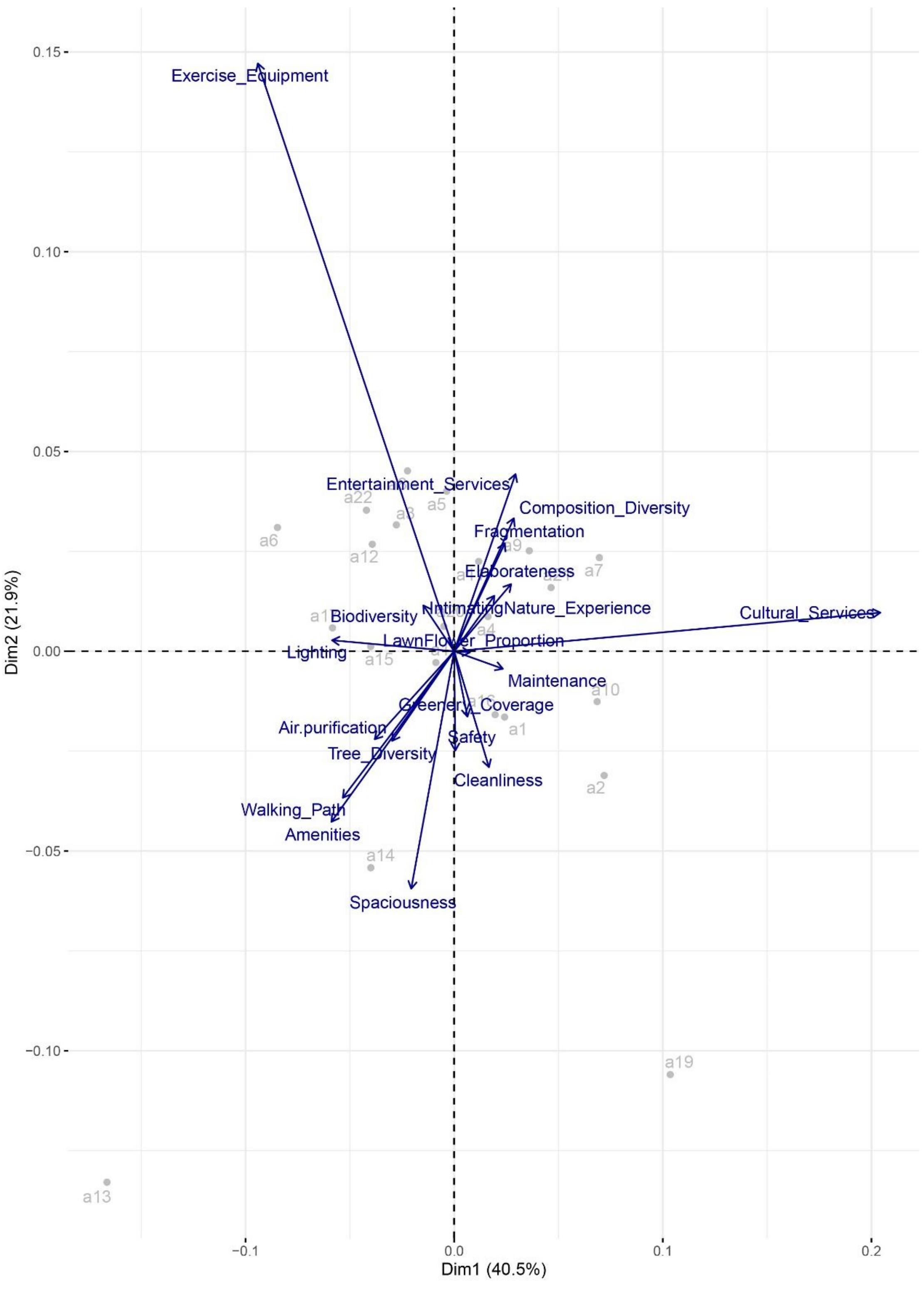

Supplementary Figure 1. Correspondence pattern of perceived park quality features with all 22 parks. Urban parks are in grey points and quality features (from Table 2) and physical, mental and social health are in blue texts. Long lines between the quality features and the origin indicate a strong association. Small angles between two lines of quality features indicate associations. Lines with angles near 180 degrees present negative associations. The distance between any urban parks shows a measure of their similarity.

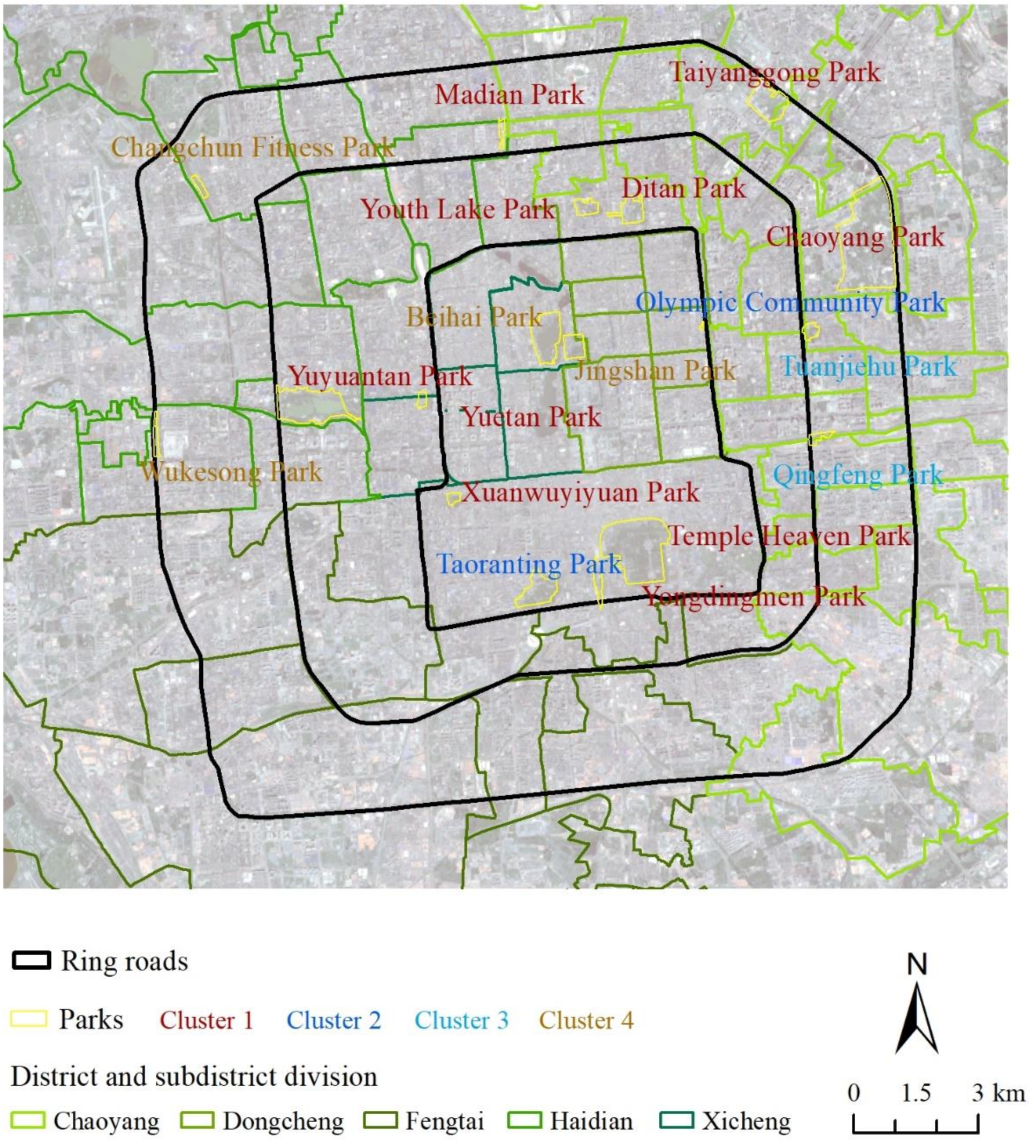

Supplementary Figure 2. The distribution of urban park clustering by the health effect of recreation from on-site survey data in 2021.

The distribution of urban park clustering by recreation health effect from data in 2021 displayed similar pattern to the results from data in 2020. First, the properties of these four clusters were still corresponding to worst social health, best social health values, highest physical and mental health scores, and moderate social, physical, or mental health conditions, respectively. Second, a majority of urban parks retained in their clusters. However, since this large sampling in 2021 was available only for 18 urban parks, there did exist slight differences, compared to the results from all 22 parks in 2020.

Supplementary Table 1. Scenes of park quality features recorded in the on-site study

Characteristics of urban park quality		Park	Landscape
Social interaction	Choir performance by community elderly	Madian Park	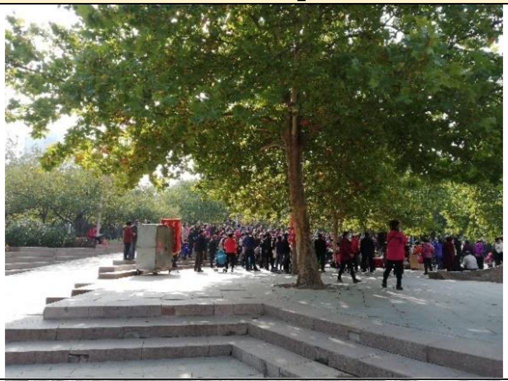
	Square-dancing by mixed-age groups	Tuanjiehu Park	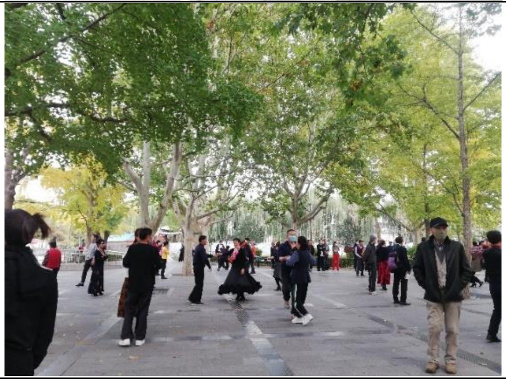

Management	Lakeside fences and greenery	Taoranting Park	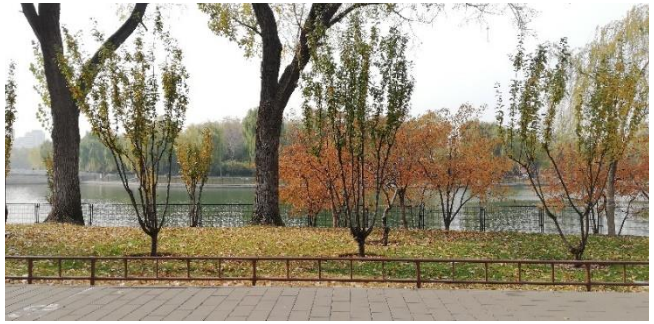
Infrastructure	Walking path and touring vehicles	Chaoyang Park	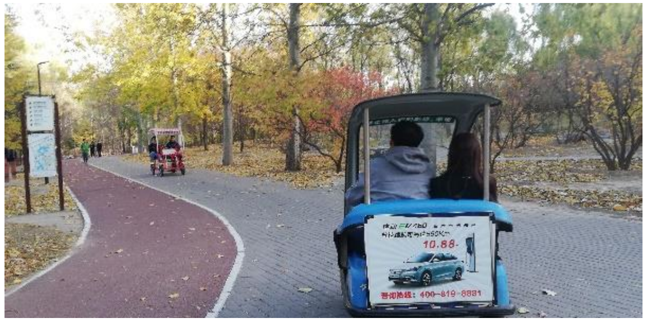
	Exercise equipment	Chaoyang Park	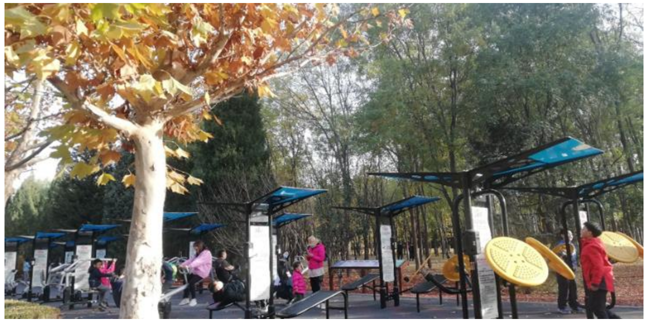

Landscape structure	Composition diversity	Youth Lake Park	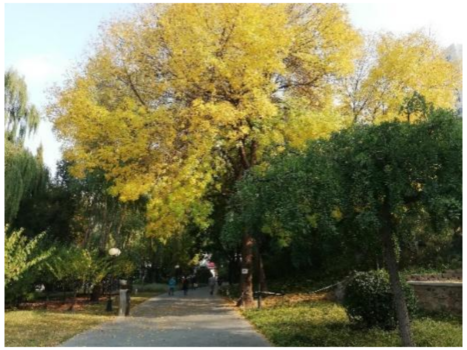
		Madian Park	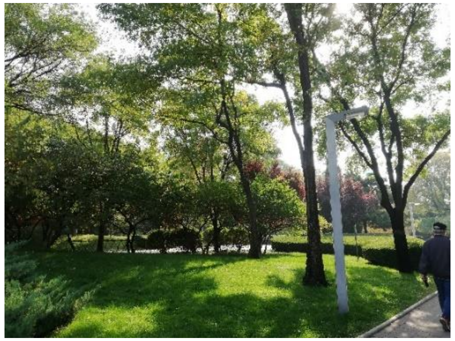

		Taiyanggong Park	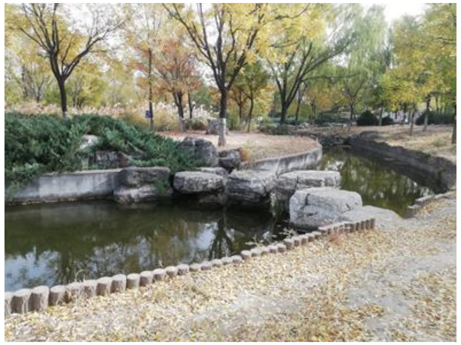
		Yongdingmen Park	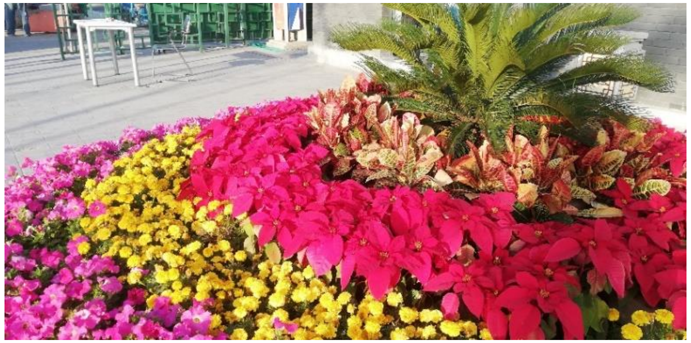

Ecosystem services	Cultural services	Tiantan Park	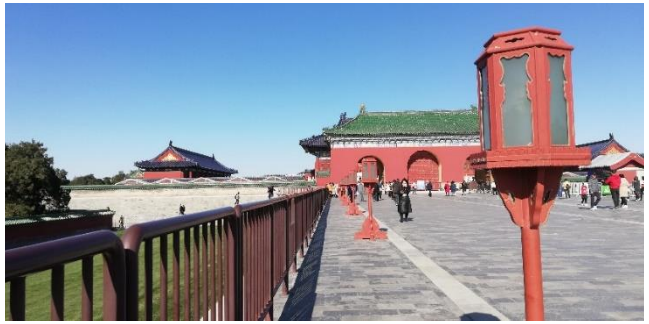
	Entertainment services	Chaoyang Park	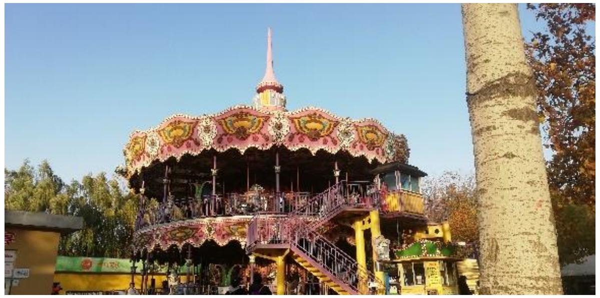

		Madian Park	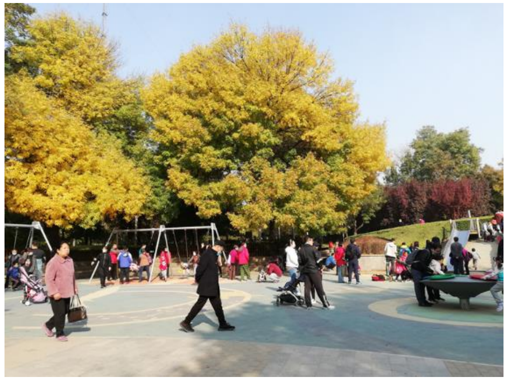
		Wanfangting Park	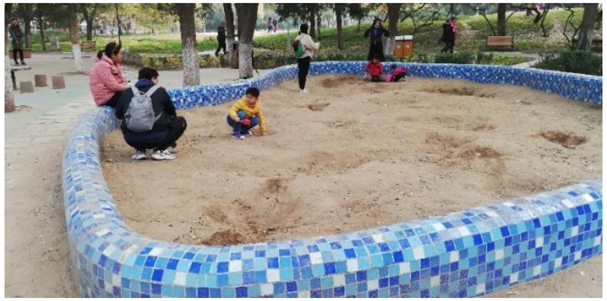

Supplementary Table 2. Comparison between groups based on demographics, accessibility, urbanization, and park type factors (two-sided Mann-Whitney U test)

Feature	Gender	Age	Access to park	Urbanization level	Park type
Physical health		**		b+c	**
Physical activity		*			
Mental health	*				
Social interaction		***			
Social trust					
Spaciousness				a+b	
Cleanliness			*	a+b	**
Safety		**		b	**
Maintenance	*			b	**
Overall quality	*		*	b	**
Walking path					
Lighting					
Exercise equipment				b	
Amenities					*
Elaborateness				b	***
Fragmentation			*	b	**
Composition diversity				b	*
Coverage of Greenery				a+b	**
Lawn and flower proportion	*		*		
Tree diversity	*	*		a+b	
Air purification		**			
Biodiversity					**
Cultural services				a+b	***
Entertainment services	*			b	**
Intimating nature experience	**			a+b	***

Note. *$P < 0.05$; **$P < 0.01$; ***$P < 0.001$.

a urban level (R2 vs R23): * for cleanliness, tree diversity, intimate nature experience; ** for greenery coverage; *** for spaciousness, cultural services.

b urban level (R2 vs R34): * for maintenance, exercise equipment, fragmentation, composition diversity, tree diversity, entertainment services, intimate nature experience; ** for spaciousness, safety, overall quality; *** for physical health, cleanliness, greenery coverage, cultural services.

c urban level (R23 vs R34): * for physical health

Supplementary Table 3. Comparison between groups based on age and gender (two-sided Mann-Whitney U test)

Feature	C1	C2	C3	C4	C5	C6
Physical health	0.270	0.383	0.008**	0.047*	0.147	0.000***
Physical activity	0.003**	0.365	0.205	0.021*	0.140	0.646
Mental health	0.030*	0.013*	0.005**	0.915	0.503	0.381
Social interaction	0.002**	0.494	0.009**	0.014*	0.538	0.039*
Social trust	0.590	0.763	0.215	0.325	0.700	0.157
Spaciousness	0.739	0.115	0.173	0.256	0.372	0.926
Cleanliness	0.107	0.067	0.020*	0.821	0.639	0.387
Safety	0.238	0.696	0.129	0.294	0.712	0.179
Maintenance	0.328	0.035*	0.052	0.391	0.389	0.938
Overall quality	0.587	0.016*	0.069	0.170	0.291	0.946
Walking path	0.809	0.283	0.723	0.563	0.857	0.753
Lighting	0.831	0.853	0.698	0.751	0.720	0.594
Exercise equipment	0.075	0.871	0.513	0.049*	0.508	0.446
Amenities	0.734	0.650	0.047*	0.924	0.058	0.020*
Elaborateness	0.784	0.576	0.495	0.816	0.467	0.230
Fragmentation	0.172	0.132	0.259	0.940	0.833	0.775
Composition diversity	0.986	0.768	0.192	0.743	0.239	0.399
Coverage of greenery	0.233	0.069	0.135	0.717	0.764	0.998
Lawn and flower proportion	0.193	0.002**	0.065	0.363	0.990	0.571
Tree diversity	0.007**	0.018*	0.001**	0.611	0.775	0.264
Air purification	0.084	0.238	0.003**	0.384	0.268	0.019*
Biodiversity	0.637	0.284	0.099	0.628	0.508	0.659
Cultural services	0.370	0.348	0.007**	0.871	0.217	0.071
Entertainment services	0.995	0.250	0.010*	0.300	0.033*	0.070
Intimate nature experience	0.619	0.062	0.002**	0.333	0.055	0.081

Note. *$P < 0.05$; **$P < 0.01$; ***$P < 0.001$.

C1 Young man v.s. older man; C2 Young man v.s. young woman

C3 Young man v.s. older woman; C4 Older man v.s. young woman

C5 Older man v.s. older woman; C6 Young woman v.s. older woman

Supplementary Table 4. Influence of demographic and park environment factors on self-reported health.

Dependent variable	Independent variable(s)		Model estimate (ß)	Standard error of ß	Wald χ^2	Sig.	Exp (ß)	95% Wald confidence interval for Exp (ß)	
Physical health	**Age**	**15-25**	1.628	.4082	15.906	.000	5.093	2.288	11.334
		**26-50**	1.219	.3308	13.586	.000	3.385	1.770	6.474
		**51-64**	1.076	.3808	7.991	.005	2.934	1.391	6.189
		**Over 65**	0[a]	.	.	.	.	.	.
Mental health	**Gender**	**Male**	-.634	.2492	6.470	.011	.531	.326	.865
		**Female**	0[a]	.	.	.	.	.	.
Social interaction	**Age**	**26-50**	-.651	.3176	4.195	.041	.522	.280	.972
		**Over 65**	0[a]	.	.	.	.	.	.

Note. Only the significant factors are included. Exp (ß) represents the OR value.

Link function: Logit.

a. This parameter is set to zero because it is redundant.

Supplementary Table 5. Influence of demographic and park environment factors on urban park quality perception

Dependent variable	Independent variable(s)		Model estimate (ß)	Standard error of ß	Wald x^2	Sig.	Exp (ß)	95% Wald confidence interval for Exp (ß)	
Spaciousness	**Urban level**	**Heavy**	.853	.3123	7.465	.006	2.770	1.340	5.723
		**Moderate**	0[a]	.	.	.	.	.	.
	**Gender**	**Male**	-.516	.2484	4.313	.038	.597	.367	.971
		**Female**	0[a]	.	.	.	.	.	.
Cleanliness	**Urban level**	**Heavy**	1.191	.2931	16.518	.000	3.292	1.853	5.847
		**Moderate**	0[a]	.	.	.	.	.	.
	**Access to park**	**1**	-.612	.2522	5.895	.015	.542	.331	.889
		**>1**	0[a]	.	.	.	.	.	.
Safety	**Age**	**15-25**	-1.122	.4074	7.590	.006	.325	.146	.723
		**26-50**	-.756	.3378	5.007	.025	.470	.220	1.006
		**Over 65**	0[a]	.	.	.	.	.	.
	**Access to park**	**1**	-.618	.2542	5.911	.015	.539	.328	.887
		**>1**	0[a]	.	.	.	.	.	.
Maintenance	**Age**	**15-25**	-.786	.3969	3.925	.048	.455	.209	.992
		**51-64**	-.821	.3782	4.713	0.30	.440	.210	.923
		**Over 65**	0[a]	.	.	.	.	.	.
	**Gender**	**Male**	-.512	.2460	4.336	.037	.599	.370	.970
		**Female**	0[a]	.	.	.	.	.	.
Overall quality	**Urban level**	**Heavy**	.651	.3084	4.450	.035	1.917	1.047	3.508
		**Moderate**	0[a]	.	.	.	.	.	.
	**Gender**	**Male**	-.579	.2493	5.388	.020	.561	.344	.914

		**Female**	0[a]	.	.	.	.	.	.
	**Access to park**	**1**	-.722	.2549	8.030	.005	.486	.295	.800
		**>1**	0[a]	.	.	.	.	.	.
Elaborateness	**Urban level**	**Heavy**	.926	.2873	10.393	.001	2.525	1.438	4.434
		**Moderate**	0[a]	.	.	.	.	.	.
Fragmentation	**Urban level**	**Heavy**	.764	.2882	7.029	.008	2.147	1.220	3.777
		**Moderate**	0[a]	.	.	.	.	.	.
	**Access to park**	**1**	-.552	.2526	4.776	.029	.576	.351	.945
		**>1**	0[a]	.	.	.	.	.	.
Greenery coverage	**Urban level**	**Heavy**	1.141	.2961	14.841	.000	3.129	1.751	5.589
		**Moderate**	0[a]	.	.	.	.	.	.
Lawn and flower proportions	**Gender**	**Male**	-.529	.2450	4.663	.031	.589	.365	.952
		**Female**	0[a]	.	.	.	.	.	.
	**Access to park**	**1**	-.542	.2491	4.742	.029	.581	.357	.947
		**>1**	0[a]	.	.	.	.	.	.
Tree diversity	**Age**	**15-25**	-.975	.4005	5.925	.015	.377	.172	.827
		**26-50**	-.968	.3315	8.534	.003	.380	.198	.727
	**Gender**	**Male**	-.533	.2475	4.633	.031	.587	.361	.953
		**Female**	0[a]	.	.	.	.	.	.
	**Access to park**	**1**	-.522	.2514	4.309	.038	.593	.363	.971
		**>1**	0[a]	.	.	.	.	.	.
Cultural services	**Urban level**	**Heavy**	2.099	.3065	46.888	.000	8.155	4.473	14.871
		**High**	.652	.3051	4.563	.033	1.919	1.055	3.490
		**Moderate**	0[a]	.	.	.	.	.	.
	**Gender**	**Male**	-.522	.2425	4.634	.031	.593	.369	.954
		**Female**	0[a]	.	.	.	.	.	.

Intimate nature experience	**Urban level**	**Heavy**	.740	.2848	6.758	.009	2.097	1.200	3.664
		**Moderate**	0[a]	.	.	.	.	.	.
	**Gender**	**Male**	-.654	.2467	7.028	.008	.520	.321	.843
		**Female**	0[a]	.	.	.	.	.	.

Note. Only the significant factors are included. Exp (ß) represents the OR value.

Link function: Logit.

a. This parameter is set to zero because it is redundant.
