# Supplementary material for: Characteristics of urban park recreation and health during early COVID-19 by on-site survey in Beijing
Source: NPJ Urban Sustain. 2023 Jun 6;3(1):31. doi: 10.1038/s42949-023-00110-3 (PMC10243239; doi:10.1038/s42949-023-00110-3)
Supplement: Supplementary file 2 — Reporting Summary [file 42949_2023_110_MOESM2_ESM.pdf]

Corresponding author(s): Yan Sun
Last updated by author(s): May 20, 2023

Reporting Summary

Nature Portfolio wishes to improve the reproducibility of the work that we publish. This form provides structure for consistency and transparency in reporting. For further information on Nature Portfolio policies, see our Editorial Policies and the Editorial Policy Checklist.

Statistics

For all statistical analyses, confirm that the following items are present in the figure legend, table legend, main text, or Methods section.

n/a	Confirmed	
☐	☒	The exact sample size (*n*) for each experimental group/condition, given as a discrete number and unit of measurement
☐	☒	A statement on whether measurements were taken from distinct samples or whether the same sample was measured repeatedly
☐	☒	The statistical test(s) used AND whether they are one- or two-sided<br>*Only common tests should be described solely by name; describe more complex techniques in the Methods section.*
☐	☒	A description of all covariates tested
☐	☒	A description of any assumptions or corrections, such as tests of normality and adjustment for multiple comparisons
☐	☒	A full description of the statistical parameters including central tendency (e.g. means) or other basic estimates (e.g. regression coefficient) AND variation (e.g. standard deviation) or associated estimates of uncertainty (e.g. confidence intervals)
☐	☒	For null hypothesis testing, the test statistic (e.g. *F*, *t*, *r*) with confidence intervals, effect sizes, degrees of freedom and *P* value noted<br>*Give P values as exact values whenever suitable.*
☒	☐	For Bayesian analysis, information on the choice of priors and Markov chain Monte Carlo settings
☒	☐	For hierarchical and complex designs, identification of the appropriate level for tests and full reporting of outcomes
☐	☒	Estimates of effect sizes (e.g. Cohen's *d*, Pearson's *r*), indicating how they were calculated

Our web collection on statistics for biologists contains articles on many of the points above.

Software and code

Policy information about availability of computer code

Data collection	data are collected by paper questionnaire survey
Data analysis	IBM SPSS Statistics 20.0, Rstudio

For manuscripts utilizing custom algorithms or software that are central to the research but not yet described in published literature, software must be made available to editors and reviewers. We strongly encourage code deposition in a community repository (e.g. GitHub). See the Nature Portfolio guidelines for submitting code & software for further information.

Data

Policy information about availability of data

All manuscripts must include a data availability statement. This statement should provide the following information, where applicable:

- Accession codes, unique identifiers, or web links for publicly available datasets
- A description of any restrictions on data availability
- For clinical datasets or third party data, please ensure that the statement adheres to our policy

The data that support the findings of this study are available from the corresponding author upon reasonable request.

Research involving human participants, their data, or biological material

Policy information about studies with human participants or human data. See also policy information about sex, gender (identity/presentation), and sexual orientation and race, ethnicity and racism.

Reporting on sex and gender	We used gender in the questionnaire design. Gender was determined based on self-reporting. All respondents were informed of the nature of the research project and what their involvement will mean. Consent has been obtained for sharing of individual-level data from all of 225 participants in the study. The study performed gender based analyses to reveal demographic differences in urban park quality perception.
Reporting on race, ethnicity, or other socially relevant groupings	No
Population characteristics	age
Recruitment	All participants are visitors who were having fun in the park. During the survey, the respondents were chosen randomly, and researchers avoided concentrated locations or distributing the questionnaires only in a large group of visitors in order to minimize bias.
Ethics oversight	College of Forestry, Beijing Forestry University, Beijing, China

Note that full information on the approval of the study protocol must also be provided in the manuscript.

Field-specific reporting

Please select the one below that is the best fit for your research. If you are not sure, read the appropriate sections before making your selection.

☐ Life sciences ☒ Behavioural & social sciences ☐ Ecological, evolutionary & environmental sciences

For a reference copy of the document with all sections, see nature.com/documents/nr-reporting-summary-flat.pdf

Behavioural & social sciences study design

All studies must disclose on these points even when the disclosure is negative.

Study description	mixed-methods case study
Research sample	Urban park visitors in Beijing, China. In the study, 55% of participants were female and 45% were male, which is representative for urban park recreation study. We referred to previously published articles on similar topics (Ekkel and de Vries, 2017; Liu et al., 2017; Ayala-Azcárraga et al., 2019; Stessens et al., 2020).
Sampling strategy	It is random sampling procedure. We designed the study by referring to previously published articles on similar topics and performed data analysis to explore the influence of sample size. Respondents were chosen randomly, and researchers avoided concentrated locations or distributing the questionnaires only in a large group of visitors in order to minimize bias. At least 10 questionnaires were completed in each of the 22 studied parks (Stessens et al., 2020).
Data collection	We used pen and paper questionnaire to collect data. Nobody was present besides the participant and the researcher during data collection.
Timing	In October and November 2020, a person-to-person on-site questionnaire was administered to gather data on park recreationists' perceptions and rating of various green space qualities. Data for verification with same survey procedure were collected from September to November in 2021
Data exclusions	We excluded the data of 10 questionnaires from the group aged 0-14, because most of those children visited the parks based on the prerequisite of their parents accompany rather than their own choice based on park recreation attraction.
Non-participation	No participants dropped out.
Randomization	Participants were not allocated into experimental groups.

Reporting for specific materials, systems and methods

We require information from authors about some types of materials, experimental systems and methods used in many studies. Here, indicate whether each material, system or method listed is relevant to your study. If you are not sure if a list item applies to your research, read the appropriate section before selecting a response.

Materials & experimental systems

n/a	Involved in the study	
☒	☐	Antibodies
☒	☐	Eukaryotic cell lines
☒	☐	Palaeontology and archaeology
☒	☐	Animals and other organisms
☒	☐	Clinical data
☒	☐	Dual use research of concern
☒	☐	Plants

Methods

n/a	Involved in the study	
☒	☐	ChIP-seq
☒	☐	Flow cytometry
☒	☐	MRI-based neuroimaging
